# Impact of Additional Active Treatment for Prostate Cancer on Health-related Quality of Life of Men: Results from the EUPROMS 2.0 1-year Follow-up Survey

**DOI:** 10.1016/j.euros.2024.11.006

**Published:** 2024-12-17

**Authors:** Lionne D.F. Venderbos, Sebastiaan Remmers, André Deschamps, John Dowling, Ernst-Günter Carl, Nuno Pereira-Azevedo, Monique J. Roobol

**Affiliations:** a Department of Urology, Erasmus MC Cancer Institute, Erasmus University Medical Center Rotterdam, Rotterdam, The Netherlands; b Europa Uomo, Antwerp, Belgium; c Department of Urology, Entre o Douro e Vouga Medical Center, Santa Maria da Feira, Portugal

## Abstract

**Background and objective:**

In 2019 and 2021, Europa Uomo initiated the Europa Uomo Patient Reported Outcome Study (EUPROMS) and the EUPROMS 2.0 survey, with the goal of collecting data on patients’ self-reported perspective on physical and mental well-being outside of a clinical trial setting, to be able to investigate the burden of prostate cancer (PCa) treatment from a patient-to-patient perspective. Acknowledging the importance of collecting quality of life (QoL) follow-up data, a 1-yr follow-up (1yrFU) study was conducted to assess the effect of additional PCa treatment on QoL.

**Methods:**

Men with PCa who participated in the EUPROMS 2.0 survey and indicated that they were open to collection of a follow-up measurement were reinvited to complete the 1yrFU survey. The EUPROMS 2.0 1yrFU survey included the validated European Quality of Life 5 Dimension 5 Level (EQ-5D-5L), European Organization for the Research and Treatment of Cancer Quality of Life Questionnaire (EORTC-QLQ-C30), Expanded Prostate Cancer Index Composite Short Form (EPIC-26), and International Index of Erectile Function (IIEF)-15 overall satisfaction domains. Descriptive statistics were used to assess demographic characteristics and to analyze the patient-reported outcome data.

**Key findings and limitations:**

A total of 1006 (54%) men completed the survey. The median age at the time of questionnaire completion was 72 yr (interquartile range 66–76 yr). Of them, 641 men (64%) underwent no new treatment, while 365 men (36%) underwent new treatment, including 247 (247/365, 68%) for PCa. In total, 114 patients (46%) underwent new androgen deprivation therapy (ADT) and 81 (33%) new external beam radiotherapy (EBRT). It is indicated that the impact of new ADT and EBRT on sexual function is immediate and detrimental, and continues to last over time. However, for men who underwent EBRT or radical prostatectomy earlier and did not undergo new treatment, slight improvements on various domains are reported.

**Conclusions and clinical implications:**

The EUPROMS 2.0 1yrFU study provides additional information on treatments that are already in common use and will help future PCa patients to make informed and shared decisions on PCa treatment.

**Patient summary:**

The follow-up data on quality of life collected by Europa Uomo can be used to inform future prostate cancer (PCa) patients about the impact of undergoing (multiple) PCa treatment(s).

## Take Home Message

Of 1006 men whom completed the EUPROMS-2.0 1-yr follow-up survey, 36.3% underwent new prostate cancer treatment, mainly new androgen deprivation therapy (46.2%) and new external beam radiotherapy (32.8%). The impact of new active treatments on sexual function is immediate and detrimental, and continues to last over time.

## 1 Introduction

Prostate cancer (PCa) is an important health problem for men. Cancer statistics indicate that in 2020 it was the second most frequent cancer type and the fifth leading cause of cancer death among men, translating into 1.4 million new cases and 375 000 deaths worldwide [1]. Europa Uomo—the PCa patient coalition in Europe—stands up for the interests of PCa patients through, among others, working to improve diagnosis, treatment, support, and quality of life (QoL; www.europa-uomo.org).

Depending on the disease stage at the time of diagnosis, there are various treatment options for PCa. All may negatively affect patients’ health-related QoL, both physically and mentally [2]. In 2019, Europa Uomo initiated the Europa Uomo Patient Reported Outcome Study (EUPROMS), and in October 2021, they launched the EUPROMS 2.0 survey [3], [4]. Both surveys had the goal of collecting data on patients’ self-reported perspective on physical and mental well-being outside of a clinical trial setting, to be able to investigate the burden of PCa treatment from a patient-to-patient perspective [3], [4]. Raising awareness for QoL after PCa diagnosis, instead of just (oncological) survival, and to provide patients with an idea of what to expect after PCa treatment were the most important motivators for Europa Uomo to initiate the EUPROMS and EUPROMS 2.0 studies. Acknowledging the importance of collecting QoL follow-up data, participants from EUPROMS 2.0 who had indicated that they were open to collection of a follow-up measurement were reinvited to complete a survey 1 yr after EUPROMS 2.0. With such additional follow-up data, it can be assessed whether QoL develops for men undergoing no new treatment and new, subsequent treatment for PCa, and if so how. It is hypothesized that any additional PCa treatment will have its effect on QoL.

## 2 Patients and methods

### 2.1 Patient screening criteria, recruitment, and data collection

In October 2021, the EUPROMS 2.0 online survey was open to men diagnosed with PCa and who were currently undergoing treatment for PCa (including active surveillance [AS]) or had undergone treatment for PCa in the past. At the end of the survey, a question was included on whether men would be willing to participate in a follow-up survey in 1 yr time. If they were interested they could fill in their e-mail addresses. For the follow-up survey, men who had left their e-mail addresses were contacted and invited to participate 1 yr later. Survey distribution and data collection were handled by Ydeal (ydeal.net) to meet both IT and legal requirements. Ydeal sent out the invitations via e-mail. The e-mail contained a link to the survey that was on a secure platform, hosted on European servers according to the ISO27001 standards. A total of four reminders were sent to the men who had not yet responded to the first or subsequent invitations to complete the survey. The survey was open between March 1 and April 10, 2023.

### 2.2 Patient-reported outcome measures

The EUPROMS 2.0 1-yr follow-up (1yrFU) survey was available in 19 languages and included validated measures to evaluate generic health (European Quality of Life 5 Dimension 5 Level [EQ-5D-5L]) [5], [6], [7], cancer-specific QoL (European Organization for the Research and Treatment of Cancer Quality of Life Questionnaire [EORTC-QLQ-C30]) [8], [9], and prostate-specific health (Expanded Prostate Cancer Index Composite Short Form [EPIC-26]) [10], [11]. Furthermore, two items of the International Index of Erectile Function 15 (IIEF-15) were added [12]. For the last employment and educational levels, we used data from the EUPROMS 2.0 survey.

The characteristics of the validated EQ-5D-5L, EORTC-QLQ-C30, and EPIC-26 have been described previously [3], [4]. In short, the EQ-5D-5L consists of five dimensions: mobility, self-care, usual activities, pain/discomfort, and anxiety depression [5], [6], [7]; each dimension has five answering options: no problems, slight, moderate, severe, and extreme problems [5], [6], [7]. The EORTC-QLQ-C30 (version 3) has five functional scales, three symptom scales, a global health status/QoL scale, and a few single items assessing additional symptoms commonly reported by cancer patients [8]. Scale scores were calculated by averaging items within scales and transforming average scores linearly. All the scale scores range from 0 to 100. For the functional scales, a higher score represents a high or healthy level of functioning. For a symptom scale, a high score represents a high level of symptomatology or problems [9]. The EPIC-26 contains five multi-item domains and a single item on overall urinary bother. All EPIC-26 domains are reported on a 0–100 scale; higher scores represent more favorable health-related QoL [10], [11]. To understand when changes in symptom burden among PCa patients and survivors are clinically relevant, Skolarus et al [13] have established the minimally important difference (MID). The MID for the urinary incontinence domain is 6–9 points, for the urinary irritative/obstructive domain 5–7 points, for the bowel domain 4–6 points, for the sexual domain 10–12 points, and for the vitality/hormonal domain 4–6 points. Questions 13 and 14 of the IIEF-15 have a 5-point Likert scale: 1 indicating very dissatisfied and 5 indicating very satisfied. Together they represent the “overall satisfaction” score. Satisfaction was categorized as follows: overall satisfaction ≥8—satisfied, overall satisfaction <8—not satisfied [12].

### 2.3 Statistical analysis

Descriptive statistics were used to assess the demographic characteristics of the men who completed the EUPROMS 2.0 1yrFU survey and to analyze the outcomes of the EQ-5D-5L, EORTC-QLQ-C30, EPIC-26, and IIEF-15 overall satisfaction scales. Health-related QoL of men who underwent new PCa treatment is stratified by new androgen deprivation therapy (ADT) or new external beam radiotherapy (EBRT) at 1yrFU. Other reported (combinations of) treatments were classified as miscellaneous due to low numbers. Furthermore, we stratified health-related QoL of men who were treated with EBRT or radical prostatectomy (RP) at the time of EUPROMS 2.0 and indicated that they did not underwent new treatment at 1yrFU. For these four groups, health-related QoL outcomes between EUPROMS 2.0 and the 1yrFU survey are described. R version 4.2.1 was used to perform all analyses [14].

## 3 Results

Of the 1879 men who were invited to complete the 1yrFU survey, 1006 (54%) completed it between March 1 and April 10, 2023. The median age of respondents at the time of questionnaire completion was 72 yr (interquartile range [IQR] 66–76; Table 1). Slightly more men with higher/intermediate managerial employment (higher managerial 26% vs 21%; intermediate managerial 38% vs 36%) and a university entrance certificate (36% vs 30%) completed the 1yrFU survey. In total, 641 (64%) men underwent no new treatment and 365 (36%) underwent new treatment. A total of 247/365 (68%) men underwent new treatment for PCa; 114/247 (46%) men underwent new ADT and 81/247 (33%) new EBRT. Of the 641 men who underwent no new treatment, 230 (36%) were treated with EBRT and 285 (45%) underwent RP at the time of EUPROMS 2.0.

**Table 1:** Respondent characteristics

Respondent characteristics (*N* = 1006)	
Age at completing questionnaire	
Total cohort (median, IQR)	72 (66–76)
Age at diagnosis (yr), *n* (%)	
<55	93 (9.3)
55–59	172 (17)
60–64	258 (26)
65–69	258 (26)
70–74	161 (16)
75–79	50 (5.0)
80+	14 (1.4)
Treatment profile of respondents for the most frequently reported treatment modalities, *n* (%)	
Men with new ADT treatment at 1-yr FU	114 (11)
Men with new EBRT treatment at 1-yr FU	81 (8.1)
Treated with EBRT at the time of EUPROMS 2.0, no new treatment at 1-yr FU	230 (23)
Treated with RP at the time of EUPROMS 2.0, no new treatment at 1-yr FU	285 (28)
Age at completing questionnaire per treatment, median (IQR)	
Men with new ADT treatment at 1-yr FU	72 (67–76)
Men with new EBRT treatment at 1-yr FU	69 (64–74)
Treated with EBRT at the time of EUPROMS 2.0, no new treatment at 1-yr FU	73 (68–77)
Treated with RP at the time of EUPROMS 2.0, no new treatment at 1-yr FU	69 (65–74)
Last employment (before retirement), *n* (%)	
Higher managerial	263 (26)
Intermediate managerial	386 (38)
Junior managerial	128 (13)
Skilled manual worker	66 (6.6)
Semiskilled manual worker	8 (0.8)
Unskilled manual worker	8 (0.8)
Unemployed	14 (1.4)
Other	133 (13)
Education, *n* (%)	
University entrance certificate	363 (36)
Entrance certificate for a higher technical college	349 (35)
Comprehensive school	97 (9.6)
Intermediate/secondary school	61 (6.1)
Lower secondary school or equivalent	26 (2.6)
Other	108 (11)
None	2 (0.2)
Country of residence, *n* (%)	
Australia	4 (0.4)
Belgium	29 (2.9)
Canada	68 (6.8)
Cyprus	3 (0.3)
Czech Republic	2 (0.2)
Denmark	51 (5.1)
Estonia	1 (0.1)
Finland	10 (1.0)
France	32 (3.2)
Germany	59 (5.9)
Greece	2 (0.2)
Hungary	2 (0.2)
Ireland	10 (1.0)
Italy	15 (1.5)
Lithuania	3 (0.3)
Norway	195 (19)
Poland	3 (0.3)
Portugal	1 (0.1)
Spain	4 (0.4)
Sweden	74 (7.4)
Switzerland	3 (0.3)
The Netherlands	289 (29)
UK	89 (8.9)
USA	40 (4.0)
Other	17 (1.7)
Comorbidities, *n* (%)	
Diabetes mellitus	24 (2.4)
Diabetes mellitus + obesity	4 (0.4)
Obesity	13 (1.3)
High blood pressure	298 (30)
High blood pressure + diabetes mellitus	47 (4.7)
High blood pressure + diabetes mellitus + obesity	16 (1.6)
High blood pressure + obesity	26 (2.6)
I don’t have any comorbidities	446 (44)
I don’t know if I have any comorbidities	51 (5.1)
None of the above, but other comorbidities	81 (8.1)

ADT = androgen deprivation therapy; EBRT = external beam radiotherapy; EUPROMS = Europa Uomo Patient Reported Outcome Study; FU = follow-up; IQR = interquartile range; RP = radical prostatectomy.

### 3.1 Generic, cancer-specific, and prostate-specific health

#### 3.1.1 EQ-5D-5L (generic health)

All men experience slightly more mobility problems. With respect to pain/discomfort, 43% of men with new ADT treatment experience no pain/discomfort versus 53% a year earlier, and 17% reported moderate pain/discomfort versus 11% the year before (Table 2). A similar pattern is observed for men who underwent new EBRT treatment, with the percentage of men reporting no pain/discomfort decreasing and the number of men reporting moderate and severe pain/discomfort increasing as compared with a year earlier. For the anxious/depressed domain, men who underwent EBRT and RP at the time of EUPROMS 2.0 and no new treatment at the 1yrFU survey indicate more often that they are not anxious/depressed (70% vs 60% for EBRT; 72% vs 67% for RP).

**Table 2:** EQ-5D-5L for specific PCa treatments

EQ-5D-5L	Men with new ADT treatment at 1-yr FU (*N* = 114)		Men with new EBRT treatment at 1-yr FU (*N* = 81)		Treated with EBRT at the time of EUPROMS 2.0, no new treatment at 1-yr FU (*N* = 230)		Treated with RP at the time of EUPROMS 2.0, no new treatment at 1-yr FU (*N* = 285)	
	EUPROMS 2.0 1-yr FU	EUPROMS 2.0	EUPROMS 2.0 1-yr FU	EUPROMS 2.0	EUPROMS 2.0 1-yr FU	EUPROMS 2.0	EUPROMS 2.0 1-yr FU	EUPROMS 2.0
Mobility, *n* (%)								
No problems	72 (63)	77 (68)	58 (72)	62 (77)	184 (80)	190 (83)	243 (85)	261 (92)
Slight problems	23 (20)	19 (17)	14 (17)	10 (12)	36 (16)	30 (13)	29 (10)	16 (5.6)
Moderate problems	10 (8.8)	11 (9.7)	2 (2.5)	6 (7.4)	8 (3.5)	6 (2.6)	10 (3.5)	5 (1.8)
Severe problems	7 (6.1)	5 (4.4)	6 (7.4)	2 (2.5)	2 (0.9)	4 (1.7)	3 (1.1)	3 (1.1)
Unable to walk	2 (1.8)	2 (1.8)	1 (1.2)	1 (1.2)	0 (0)	0 (0)	0 (0)	0 (0)
Self-care, *n* (%)								
No problems	102 (90)	106 (93)	73 (90)	75 (93)	222 (97)	221 (96)	275 (97)	278 (98)
Slight problems	6 (5.3)	3 (2.6)	3 (3.7)	3 (3.7)	7 (3.0)	7 (3.0)	10 (3.5)	7 (2.5)
Moderate problems	5 (4.4)	3 (2.6)	4 (4.9)	1 (1.2)	1 (0.4)	2 (0.9)	0 (0)	0 (0)
Severe problems	1 (0.9)	2 (1.8)	1 (1.2)	2 (2.5)	0 (0)	0 (0)	0 (0)	0 (0)
Unable to wash/dress myself	0 (0)	0 (0)	0 (0)	0 (0)	0 (0)	0 (0)	0 (0)	0 (0)
Usual activities, *n* (%)								
No problems	60 (53)	62 (54)	50 (62)	51 (63.0)	184 (80)	175 (76)	240 (84)	236 (83)
Slight problems	30 (26)	29 (25)	18 (22)	20 (25)	36 (16)	41 (18)	34 (12)	36 (13)
Moderate problems	18 (16)	16 (14)	7 (8.6)	6 (7.4)	10 (4.3)	13 (5.7)	10 (3.5)	10 (3.5)
Severe problems	5 (4.4)	6 (5.3)	6 (7.4)	4 (4.9)	0 (0)	0 (0)	1 (0.4)	3 (1.1)
Unable to do usual activities	1 (0.9)	1 (0.9)	0 (0)	0 (0)	0 (0)	1 (0.4)	0 (0)	0 (0)
Pain/discomfort, *n* (%)								
No pain/discomfort	49 (43.0)	60 (53)	31 (38)	41 (51)	132 (57)	132 (57)	171 (60)	197 (69)
Slight pain/discomfort	41 (36.0)	38 (33)	29 (36)	29 (36)	73 (32)	73 (32)	97 (34)	70 (25)
Moderate pain/discomfort	19 (17)	12 (11)	14 (17)	7 (8.6)	19 (8.3)	18 (7.8)	15 (5.3)	17 (6.0)
Severe pain/discomfort	5 (4.4)	4 (3.5)	7 (8.6)	4 (4.9)	5 (2.2)	6 (2.6)	2 (0.7)	1 (0.4)
Extreme pain/discomfort	0 (0)	0 (0)	0 (0)	0 (0)	1 (0.4)	1 (0.4)	0 (0)	0 (0)
Anxious/depressed, *n* (%)								
Not anxious/depressed	60 (53)	56 (49)	40 (49)	39 (48)	160 (70)	139 (60)	205 (72)	190 (67)
Slightly anxious/depressed	28 (25)	32 (28)	26 (32)	21 (26)	53 (23)	65 (28)	57 (20)	72 (25)
Moderately anxious/depressed	20 (18)	17 (15)	10 (12)	14 (17)	16 (7.0)	19 (8.3)	19 (6.7)	17 (6.0)
Severely anxious/depressed	5 (4.4)	6 (5.3)	5 (6.2)	6 (7.4)	1 (0.4)	7 (3.0)	3 (1.1)	5 (1.8)
Extremely anxious/depressed	1 (0.9)	3 (2.6)	0 (0)	1 (1.2)	0 (0)	0 (0)	1 (0.4)	1 (0.4)

ADT = androgen deprivation therapy; EBRT = external beam radiotherapy; EQ-5D-5L = European Quality of Life 5 Dimension 5 Level; EUPROMS = Europa Uomo Patient Reported Outcome Study; FU = follow-up; PCa = prostate cancer; RP = radical prostatectomy.

#### 3.1.2 EORTC-QLQ-C30 (cancer-specific health)

In line with the changes on the pain/discomfort domain of the EQ-5D-5L, pain scores for men who underwent new ADT or EBRT were increased (Table 3). For men treated with EBRT or radiotherapy at the time of EUPROMS 2.0 and no new treatment 1 yr later, the scores of the emotional and/or social scales have improved.

**Table 3:** EORTC-QLQ-C30 scale scores (median, IQR) for specific PCa treatments

	Men with new ADT treatment at 1-yr FU (*N* = 114)		Men with new EBRT treatment at 1-yr FU (*N* = 81)		Treated with EBRT at the time of EUPROMS 2.0, no new treatment at 1-yr FU (*N* = 230)		Treated with RP at the time of EUPROMS 2.0, no new treatment at 1-yr FU (*N* = 285)	
	EUPROMS 2.0 1-yr FU	EUPROMS 2.0	EUPROMS 2.0 1-yr FU	EUPROMS 2.0	EUPROMS 2.0 1-yr FU	EUPROMS 2.0	EUPROMS 2.0 1-yr FU	EUPROMS 2.0
Functional scales a, median (IQR)								
Physical	86.7 (66.7–93.3)	86.7 (73.3–93.3)	86.7 (73.3–93.3)	93.3 (80–100)	93.3 (86.7–100)	93.3 (86.7–100)	100 (86.7–100)	100 (93.3–100)
Role	83.3 (66.7–100)	100 (66.7–100)	100 (66.7–100)	100 (66.7–100)	100 (83.3–100)	100 (83.3–100)	100 (100–100)	100 (100–100)
Cognitive	83.3 (66.7–100)	83.3 (66.7–100)	83.3 (66.7–100)	83.3 (83.3–100)	83.3 (83.3–100)	83.3 (83.3–100)	100 (83.3–100)	100 (83.3–100)
Emotional	83.3 (58.3–91.7)	75 (58.3–91.7)	83.3 (66.7–91.7)	83.3 (66.7–91.7)	91.7 (75.0–100)	91.7 (75.0–100)	100 (75–100)	91.7 (75–100)
Social	83.3 (50–100)	83.3 (66.7–100)	83.3 (50–100)	83.3 (66.7–100)	100 (83.3–100)	83.3 (66.7–100)	100 (83.3–100)	83.3 (66.7–100)
Symptom scales b, median (IQR)								
Fatigue	33.3 (22.2–55.6)	33.3 (22.2–44.4)	33.3 (22.2–55.6)	33.3 (11.1–33.3)	22.2 (0–33.3)	22.2 (0–33.3)	11.1 (0–33.3)	11.1 (0–33.3)
Pain	8.3 (0–33.3)	0 (0–16.7)	16.7 (0–33.3)	0 (0–33.3)	0 (0–16.7)	0 (0–16.7)	0 (0–16.7)	0 (0–16.7)
Nausea and vomiting	0 (0–0)	0 (0–0)	0 (0–0)	0 (0–0)	0 (0–0)	0 (0–0)	0 (0–0)	0 (0–0)
Single items c, median (IQR)								
Dyspnea	33.3 (0–33.3)	0 (0–33.3)	0 (0–33.3)	0 (0–33.3)	0 (0–33.3)	0 (0–33.3)	0 (0–33.3)	0 (0–0)
Loss of appetite	0 (0–0)	0 (0–0)	0 (0–0)	0 (0–0)	0 (0–0)	0 (0–0)	0 (0–0)	0 (0–0)
Insomnia	33.3 (0–33.3)	33.3 (0–33.3)	33.3 (0–33.3)	33.3 (0–66.7)	0 (0–33.3)	33.3 (0–33.3)	0 (0–33.3)	0 (0–33.3)
Constipation	0 (0–33.3)	0 (0–33.3)	0 (0–33.3)	0 (0–33.3)	0 (0–33.3)	0 (0–33.3)	0 (0–0)	0 (0–0)
Diarrhea	0 (0–25)	0 (0–33.3)	0 (0–33.3)	0 (0–0)	0 (0–0)	0 (0–33.3)	0 (0–0)	0 (0–0)
Financial difficulties	0 (0–0)	0 (0–0)	0 (0–0)	0 (0–0)	0 (0–0)	0 (0–0)	0 (0–0)	0 (0–0)
Global health status c, median (IQR)	75 (58.3–83.3)	75 (58.3–83.3)	83.3 (58.3–83.3)	75 (58.3–83.3)	83.3 (75–91.7)	83.3 (75–91.7)	93.1 (86.5–97.4)	92.7 (86–97.4)

ADT = androgen deprivation therapy; EBRT = external beam radiotherapy; EORTC-QLQ-C30 = European Organization for the Research and Treatment of Cancer Quality of Life Questionnaire; EUPROMS = Europa Uomo Patient Reported Outcome Study; FU = follow-up; IQR = interquartile range; RP = radical prostatectomy.

a Functional scales/global health status: a higher score indicates better functioning/better quality of life.

b Symptom scales: a higher score means more symptoms, worse functioning.

c Single items: a higher score means more symptoms, worse functioning.

#### 3.1.3 EPIC-26 (prostate-specific health)

##### 3.1.3.1 Urinary function

**Fig. 1:**
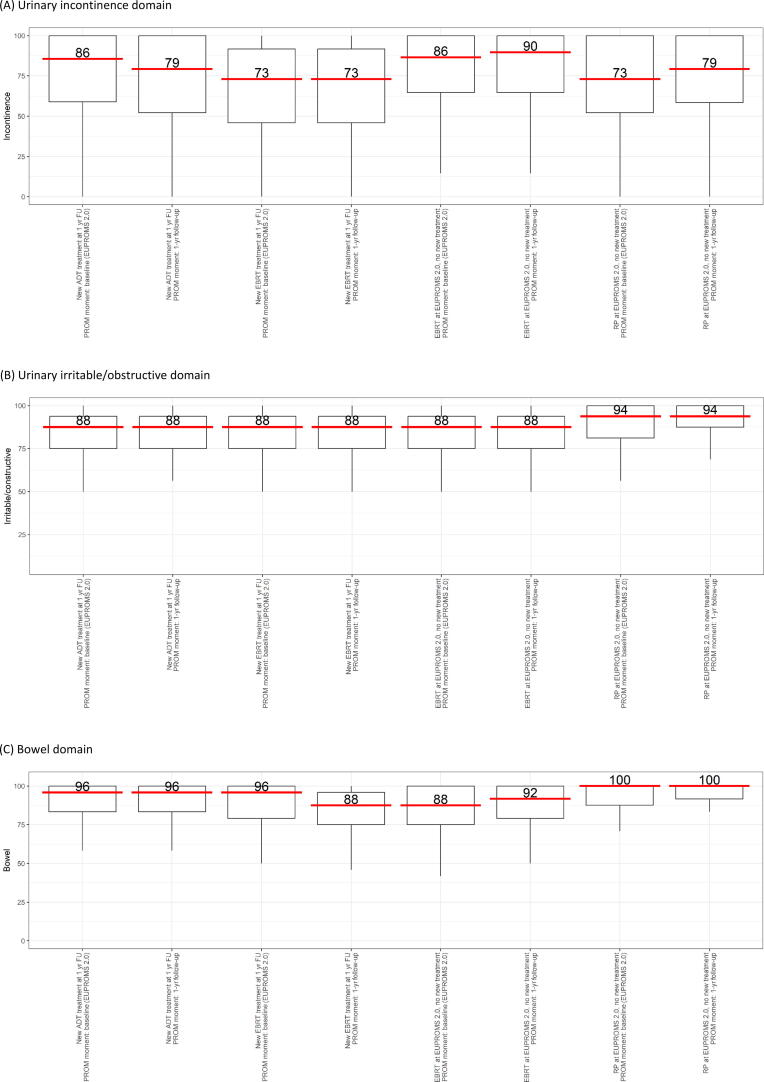

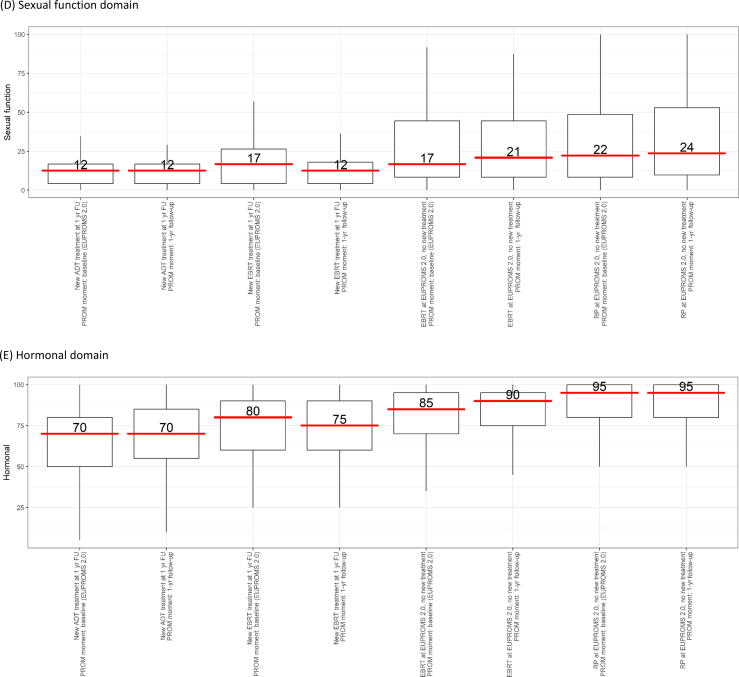
EPIC-26 domain scores for specific PCa treatments: (A) urinary incontinence domain, (B) urinary irritable/obstructive domain, (C) bowel domain, (D) sexual function domain, and (E) hormonal domain. All domains are reported on a 0–100 scale, with higher scores representing favorable HRQoL. ADT = androgen deprivation therapy; EBRT = external beam radiotherapy; EPIC-26 = Expanded Prostate Cancer Index Composite Short Form; EUPROMS = Europa Uomo Patient Reported Outcome Study; FU = follow-up; HRQoL = health-related quality of life; PCa = prostate cancer; PROM = patient-reported outcome measure; RP = radical prostatectomy.

The urinary incontinence domain score has decreased from 86 to 79 for men who underwent new ADT (Fig. 1A). While for men who were treated with EBRT or RP at the time of EUPROMS 2.0 and no new treatment 1 yr later, the score improved (90 vs 87 for EBRT; 79 vs 73.0 for RP). Both the decrease in score for men who underwent new ADT (–6.2 points) and the increase in score for men who were treated with RP at the time of EUPROMS 2.0 (+6.3 points) fall within the MID range for urinary incontinence (6–9 points). With respect to pad use, 65% of men who underwent new ADT use no pads (35% use one or more pads) versus 75% of men who used no pads a year earlier (25% used ≥1 pad; Table 4). Of the men who were treated with RP at the time of EUPROMS 2.0, 61% report using no pads at 1yrFU versus 57% at the time of EUPROMS 2.0. Among men who underwent EBRT at the time of EUPROMS, the percentage of men using two pads per day increased (6% vs 3%).

**Table 4:** EPIC-26 individual item scores (*N*, %) for specific PCa treatments

	Men with new ADT treatment at 1-yr FU (*N* = 114)		Men with new EBRT treatment at 1-yr FU (*N* = 81)		Treated with EBRT at the time of EUPROMS 2.0, no new treatment at 1-yr FU (*N* = 230)		Treated with RP at the time of EUPROMS 2.0, no new treatment at 1-yr FU (*N* = 285)	
	EUPROMS 2.0 1-yr FU	EUPROMS 2.0	EUPROMS 2.0 1-yr FU	EUPROMS 2.0	EUPROMS 2.0 1-yr FU	EUPROMS 2.0	EUPROMS 2.0 1-yr FU	EUPROMS 2.0
How many pads or adult diapers per day did you usually use to control leakage during the last 4 wk? *N* (%)								
None	74 (65)	85 (75)	43 (53)	49 (61)	174 (76)	178 (77)	174 (61)	161 (57)
1 pad per day	26 (23)	17 (15)	24 (30)	21 (26)	33 (14)	35 (15)	76 (27)	81 (28)
2 pads per day	5 (4.4)	6 (5.3)	6 (7.4)	7 (8.6)	14 (6.1)	6 (2.6)	20 (7.0)	22 (7.7)
3 or more pads per day	9 (7.9)	6 (5.3)	8 (9.9)	4 (4.9)	9 (3.9)	11 (4.8)	15 (5.3)	21 (7.4)
How big a problem, if any, has losing control of your stools been for you? *N* (%)								
No problem	90 (79)	85 (75)	58 (72)	58 (72)	174 (76)	156 (68)	256 (90)	257 (90)
Very small problem	9 (7.9)	13 (11)	11 (14)	13 (16)	32 (14)	44 (19)	22 (7.7)	19 (6.7)
Small problem	6 (5.3)	6 (5.3)	5 (6.2)	4 (4.9)	13 (5.7)	18 (7.8)	4 (1.4)	5 (1.8)
Moderate problem	5 (4.4)	5 (4.4)	3 (3.7)	4 (4.9)	6 (2.6)	8 (3.5)	3 (1.1)	3 (1.1)
Big problem	4 (3.5)	5 (4.4)	4 (4.9)	2 (2.5)	5 (2.2)	4 (1.7)	0 (0)	1 (0.4)
How big a problem, if any, have bloody stools been for you? *N* (%)								
No problem	108 (95)	104 (91)	71 (88)	76 (94)	196 (85)	192 (84)	275 (97)	275 (97)
Very small problem	4 (3.5)	5 (4.4)	4 (4.9)	3 (3.7)	21 (9.1)	21 (9.1)	6 (2.1)	7 (2.5)
Small problem	1 (0.9)	4 (3.5)	4 (4.9)	1 (1.2)	7 (3.0)	5 (2.2)	2 (0.7)	1 (0.4)
Moderate problem	1 (0.9)	0 (0)	2 (2.5)	0 (0)	5 (2.2)	9 (3.9)	0 (0)	1 (0.4)
Big problem	0 (0)	1 (0.9)	0 (0)	1 (1.2)	1 (0.4)	3 (1.3)	2 (0.7)	1 (0.4)
Overall, how big a problem have your bowel habits been for you during the last 4 wk? *N* (%)								
No problem	65 (57)	67 (59)	38 (47)	44 (54)	122 (53)	112 (49)	209 (73)	203 (71)
Very small problem	28 (25)	29 (25)	23 (28)	22 (27)	62 (27)	64 (28)	46 (16)	56 (20)
Small problem	11 (9.7)	6 (5.3)	9 (11)	6 (7.4)	28 (12)	30 (13)	21 (7.4)	14 (4.9)
Moderate problem	6 (5.3)	10 (8.8)	8 (9.9)	8 (9.9)	14 (6.1)	17 (7.4)	9 (3.2)	11 (3.9)
Big problem	4 (3.5)	2 (1.8)	3 (3.7)	1 (1.2)	4 (1.7)	7 (3.0)	0 (0)	1 (0.4)
How would you describe the usual quality of your erections during the last 4 wk? *N* (%)								
None at all	102 (90)	92 (81)	60 (74)	53 (65)	104 (45)	102 (44)	114 (40)	117 (41)
Not firm enough for any sexual activity	7 (6.1)	6 (5.3)	7 (8.6)	11 (14)	42 (18)	42 (18)	52 (18)	52 (18)
Firm enough for masturbation and foreplay only	3 (2.6)	12 (11)	10 (12)	10 (12)	54 (24)	61 (27)	73 (26)	73 (26)
Firm enough for intercourse	2 (1.8)	4 (3.5)	4 (4.9)	7 (8.6)	30 (13)	25 (11)	46 (16)	43 (15)
Overall, how big a problem has your sexual function or lack of sexual function been for you during the last 4 wk? *N* (%)								
No problem	37 (33)	33 (29)	19 (24)	16 (20)	37 (16)	33 (14)	42 (15)	35 (12)
Very small problem	14 (12)	14 (12)	10 (12)	10 (12)	37 (16)	34 (15)	47 (17)	39 (14)
Small problem	11 (9.7)	12 (11)	10 (12)	12 (15)	48 (21)	41 (18)	48 (17)	53 (19)
Moderate problem	18 (16)	22 (19)	19 (24)	17 (21)	55 (24)	57 (25)	70 (25)	81 (28)
Big problem	34 (30)	33 (29)	23 (28)	26 (32)	53 (23)	65 (28)	78 (27)	77 (27)

ADT = androgen deprivation therapy; EBRT = external beam radiotherapy; EPIC-26 = Expanded Prostate Cancer Index Composite Short Form; EUPROMS = Europa Uomo Patient Reported Outcome Study; FU = follow-up; PCa = prostate cancer; RP = radical prostatectomy.

##### 3.1.3.2 Bowel function

A decrease in the median bowel domain score was seen for men who underwent new EBRT (88 vs 96; –8.3 points = clinically relevant), while for men who underwent EBRT at the time of EUPROMS 2.0, a slight increase in score was seen (92–88, +4.2 = clinically relevant; Fig. 1C).

##### 3.1.3.3 Sexual function

The sexual function domain scores for new ADT and new EBRT were already low at the time of EUPROMS 2.0 and remained low at 1yrFU (new ADT 13 [IQR 4.2–17]; new EBRT 13 [IQR 4.2–18]; Fig. 1D). For men who underwent treatment at the time of EUPROMS 2.0 and no new treatment 1 yr later, a slight improvement is seen (earlier EBRT 21 [IQR 8.3–45] vs 17 [IQR 8.3–45]; earlier RP 24 [IQR 9.7–53] vs 22 [IQR 8.3–49]). The improvement is, however, not in the clinically relevant range of a 10–12-point change.

At 1yrFU, men undergoing new ADT treatment more often reported to have no erection (90%) compared with the new EBRT (74%), earlier EBRT (45%), and earlier RP (40%) groups (Table 4). A lack of sexual function is experienced as a moderate/big problem by 46% of new ADT men, 52% of new EBRT men, 47% of earlier EBRT men, and 52% of earlier RP men.

**Fig. 2:**
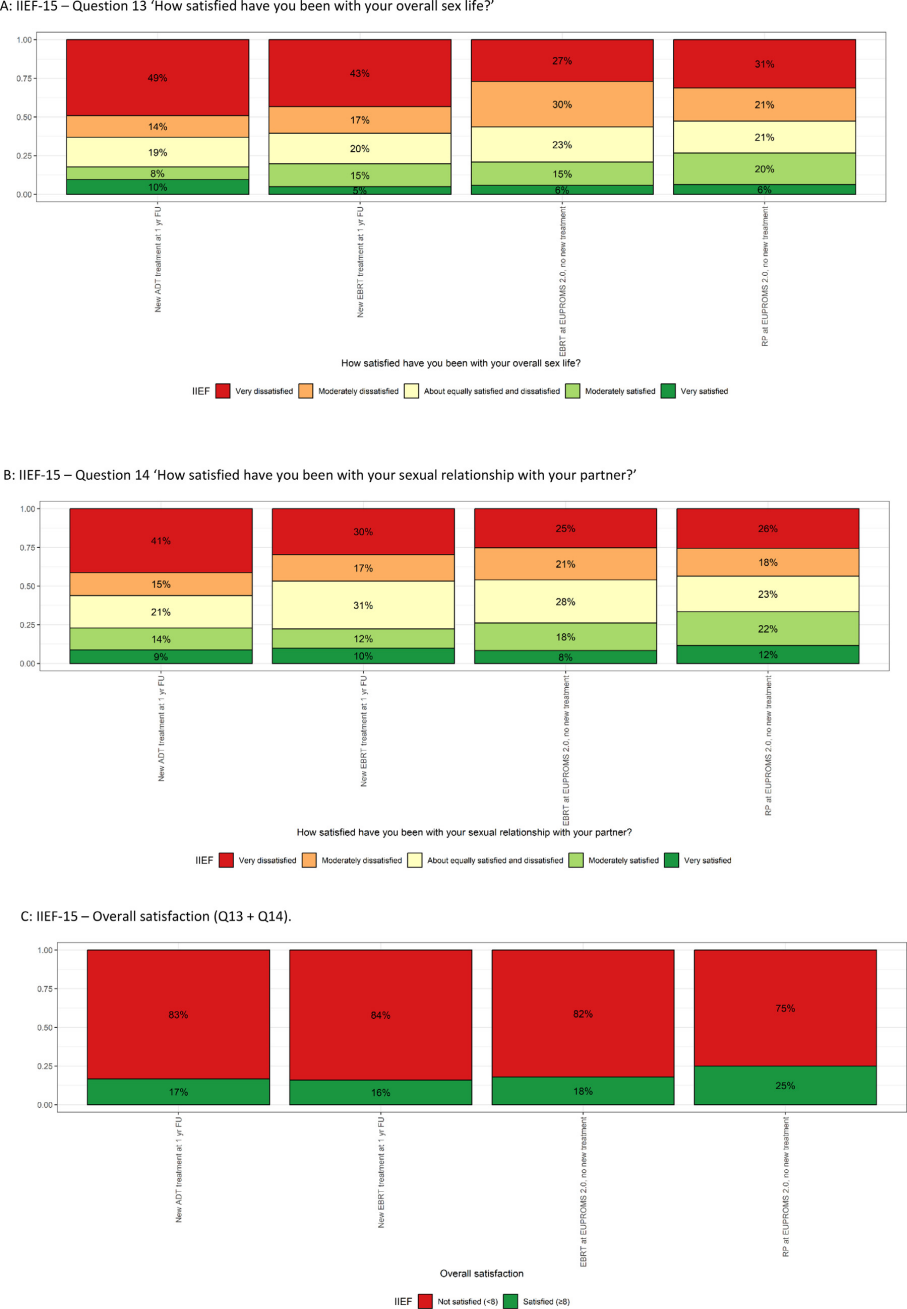
IIEF-15 overall satisfaction domain (Q13 + Q14) score for specific PCa treatments. (A) IIEF-15—question 13: “How satisfied have you been with your overall sex life?’ (B) IIEF-15—question 14: “How satisfied have you been with your sexual relationship with your partner?” (C) IIEF-15—overall satisfaction (Q13 + Q14). ADT = androgen deprivation therapy; EBRT = external beam radiotherapy; EUPROMS = Europa Uomo Patient Reported Outcome Study; FU = follow-up; IIEF-15 = International Index of Erectile Function 15; PCa = prostate cancer; RP = radical prostatectomy.

Using two questions from the IIEF-15, satisfaction with the overall sex life of men and the sexual relationship with their partner has been assessed. Regarding the satisfaction with the sexual relationship with the partner, 56% of new ADT, 47% of new EBRT, 46% of earlier EBRT, and 44% of earlier RP men are moderately/very dissatisfied, while 23%, 22%, 26%, and 33% are moderately/very satisfied, respectively (Fig. 2). Overall, 75–84% of men are not satisfied with their sex life.

## 4 Discussion

The EUPROMS 2.0 study has continued its data collection by inviting men who participated before and agreed to complete a 1yrFU survey. Of the 1006 men who completed the survey, 36% underwent new PCa treatment, mainly new ADT (46%) and new EBRT (33%). The data collected indicate that the impact of new active treatments on sexual function is immediate and detrimental, and continues to last over time. However, for men who underwent EBRT or RP earlier and did not undergo new treatment, slight improvements in various domains are reported as well. The results of the EUPROMS 2.0 1yrFU study provide additional information on treatments that are already in common use.

With the EUPROMS studies (EUPROMS 1.0, EUPROMS 2.0, and EUPROMS 2.0 1yrFU), Europa Uomo, as a patient powered research network, has collected real-world data from a patient-to-patient perspective as a complementary source of randomized controlled trial (RCT) data for establishing a more robust evidence base on the health-related QoL effects of PCa treatment [15], [16]. The surveys have shown that the collection of electronic patient-reported outcomes (ePROs) in an older patient population (median age in EUPROMS 2.0 1yrFU is 71.5 yr [IQR 66–76 yr]) is possible and provides knowledge about how PCa (treatment) affects the more aging population [17]. Owing to the reduction of serum testosterone levels induced by ADT, men who underwent new ADT in the EUPROMS 2.0 1yrFU study report symptoms of fatigue, insomnia, and erectile dysfunction, as well as the lowest EORTC-QLQ-C30 global health status score (compared with the other treatments in our study). This is in line with the results reported in the literature [18]. Men undergoing new EBRT had an 8.3 lower median EPIC-26 bowel domain score, which is a clinically relevant decline. However, for men who underwent EBRT before and who did not underwent new PCa treatment, it is seen that the bowel domain score increases again (clinically relevant +4.2 points). For the EPIC-26 hormonal domain, a similar clinically relevant pattern is seen. For sexual function, however, the increase and decrease in scores are not clinically relevant.

When working with ePROs (similar to with paper-based questionnaires), ensuring a high response rate is of vital importance for the reliability and validity of a study. In a qualitative semistructured interview study published in 2023, Dorner Østergaard et al [17] looked into the experiences of older PCa patients (average age 76 yr, range 72–80 yr) in completing ePRO documents about their QoL in a clinical trial setting, and what motivates and demotivates them. From 13 interviews with male PCa patients who underwent AS/watchful waiting, surgery, radiation, or antihormonal treatment, five main themes were identified: the ePRO frame is feasible, men thought that it was challenging to rate one’s life on a scale, men got an increased disease insight, men had unmet expectations of emotional support, and men could go from motivation to demotivation [17]. Study participants indicated that they were motivated to fill out patient-reported outcome measures (PROMs) because of the idea of helping with new knowledge and because accessing and completing electronic PROM (ePROM) assessments from home were found to be easy. Furthermore, they were motivated by the new knowledge they gained through the use of ePROs about their symptoms and the possibility to follow their own progress. Men could, for instance, recognize that they were doing better 12 mo after surgery than shortly after surgery. It was, however, also indicated that, when relating to one’s own QoL, this creates an expectation that doctors and nurses will do the same in their treatment, even though it was communicated upfront that they would not receive a reply. When it turned out that this did not happen, this initial motivation could turn into demotivation as ePRO knowledge was not actually used to tailor their treatment and follow-up [17].

So far, many men have participated in and completed one or more of the EUPROMS surveys. Sharing information on your QoL may help future men who are diagnosed with PCa to choose a treatment that fits their needs and expectations, which can serve as a motivator to participate. Subsequently, men who have completed an ePROM questionnaire may feel empowered and better equipped to handle the symptoms and various side effects of treatment. Furthermore, they often have an improved ability to verbalize their needs [17]. This is an important aspect in the context of health literacy. Health literacy can be described as an “individual’s capacity to access, understand, communicate, evaluate, utilize, and make decisions based on health information” [19]. The importance of health literacy has been well established throughout the health care sector. Poor health literacy has been associated with negative health outcomes, inadequate use of health services, and increased occurrences of adverse events [20], [21], [22]. On the contrary, improved health literacy helps men increase their understanding of the disease and their ability to use information related to their PCa [23].

While many men have participated in the EUPROMS studies, many others decided not to contribute to the EUPROMS 1.0 and 2.0 studies, did not want to be contacted for a follow-up survey, or did not complete the EUPROMS 1yrFU survey (936/1879 [50%]). This can be recognized as a limitation of the study. Inclusion of all these men, if possible, might lead to the contribution of additional perspectives. This would help Europa Uomo in its mission to direct toward a better patient-doctor relationship, offer patients access to responsible information, and provide them with a better understanding of their disease. It should be recognized that although PROMs have been designed to narrow down the discordance in symptom perception between patients and health care providers, an inherent aspect is that what patients report on their own health status could be interpreted differently by health care providers.

The recognition of the importance of QoL follow-up data can be seen as a strength of the EUPROMS 1yrFU study. Europa Uomo has been able to mobilize a large group of men to complete a 1yrFU survey. Results of both men who underwent no new treatment for PCa and those who underwent new treatment (ie, new ADT and new EBRT) can be used for informed and shared decision-making for PCa treatment in other men diagnosed and treated for PCa.

## 5 Conclusions

In conclusion, with the EUPROMS 2.0 1yrFU study, Europa Uomo has been able to collect real-world data from a patient-to-patient perspective as a complementary source of RCT data for establishing a more robust evidence base on the health-related QoL effects of PCa treatment. Of the 1006 men who completed the survey, 36% underwent new PCa treatment, mainly new ADT (46%) and new EBRT (33%). The data collected indicate that the impact of new active treatments on sexual function is immediate and detrimental, and continues to last over time. However, for men who underwent EBRT or RP earlier and did not undergo new treatment, slight improvements in various domains are reported. The results of the EUPROMS 2.0 1yrFU study provide additional information on treatments that are already in common use and will help future PCa patients to make informed and shared decisions on which treatment to start for PCa.

  ***Author contributions*:** Lionne D.F. Venderbos had full access to all the data in the study and takes responsibility for the integrity of the data and the accuracy of the data analysis.

  *Study concept and design*: Venderbos, Remmers, Deschamps, Pereira-Azevedo, Roobol.

*Acquisition of data*: Deschamps, Dowling, Carl, Pereira-Azevedo.

*Analysis and interpretation of data*: Remmers, Venderbos, Roobol, Deschamps.

*Drafting of the manuscript*: Venderbos.

*Critical revision of the manuscript for important intellectual content*: Remmers, Deschamps, Dowling, Carl, Pereira-Azevedo, Roobol.

*Statistical analysis*: Remmers.

*Obtaining funding*: Deschamps.

*Administrative, technical, or material support*: Europa Uomo.

*Supervision*: Roobol.

*Other*: None.

  ***Financial disclosures:*** Lionne D.F. Venderbos certifies that all conflicts of interest, including specific financial interests and relationships and affiliations relevant to the subject matter or materials discussed in the manuscript (eg, employment/affiliation, grants or funding, consultancies, honoraria, stock ownership or options, expert testimony, royalties, or patents filed, received, or pending), are the following: None.

  ***Funding/Support and role of the sponsor***: The EUPROMS 2.0 1-yr follow-up study, initiated by Europa Uomo, was supported by Astellas, Amgen, AAA, Ipsen, Bayer, AstraZeneca, and Eli Lilly. The funders did not play any role in the study design, collection, analysis, or interpretation of data, or in the drafting of this paper.
